# The Effects of Kinesiotape Applied to the Lateral Aspect of the Ankle: Relevance to Ankle Sprains – A Systematic Review

**DOI:** 10.1371/journal.pone.0124214

**Published:** 2015-06-23

**Authors:** Brendan Wilson, Andrea Bialocerkowski

**Affiliations:** Menzies Health Institute Queensland/ School of Allied Health Sciences, Gold Coast Campus, Griffith University, Queensland, Australia; Bern University of Applied Sciences, SWITZERLAND

## Abstract

**Objective:**

To identify, evaluate and synthesise evidence on the effect of kinesiotape applied to the lateral aspect of the ankle, through a systematic review of quantitative studies.

**Data Sources:**

A search for quantitative studies was undertaken using key terms of “kinesiotape” and “ankle” in seven electronic databases, using the maximum date ranges. Databases included: the Cochrane Library, Cumulative Index to Nursing and Allied Health Literature, Medline, Physiotherapy Evidence Database, Scopus, SPORTDiscus and Web of Science.

**Study Selection:**

Database hits were evaluated against explicit inclusion criteria. From 107 database hits, 8 quantitative studies were included.

**Data Extraction:**

Two independent reviewers appraised the methodological rigour of the studies using the McMaster Critical Review Form for Quantitative Studies. Data were extracted on participant characteristics, kinesiotape parameters, comparison interventions, outcome measures and findings.

**Data Syntheses:**

Most studies (n=7) had good to very good methodological rigour. Meta-analysis was not possible due to heterogeneity in participants, interventions and outcome measures. No adverse events were reported. Kinesiotape may produce different effects in healthy and injured ankles. In healthy ankles, kinesiotape may increase postural control, whereas in injured ankles it may improve proprioception, plantarflexor endurance and the performance of activities. These trends were identified from a small body of evidence including 276 participants.

**Conclusions:**

It is recommended that kinesiotape may be used in clinical practice to prevent lateral ankle injuries (through its effects on postural control) and manage lateral ankle injuries due to its positive effects on proprioception, muscle endurance and activity performance. It appears that kinesiotape may not provide sufficient mechanical support to improve postural control in unstable ankles. Adverse events associated with kinseiotape are unlikely.

## Introduction

The ankle is among the most frequently injured joints during athletic activity, accounting for approximately 30% of all sports related injuries [1,2]. The most frequent ankle injury is a ligament sprain with up to 85% involving the lateral ligament complex [3]. This occurs from an inversion, supination and plantar flexion mechanism of injury [3]. People who participate in court games, team sports, contact sports, indoor sports and jumping sports are at the greatest risk of injury [1,4], and females, children and adolescents report a higher incidence of lateral ankle sprains compared with males and adults [5].

While recovery from an ankle sprain is often rapid [6], appropriate management is imperative to reduce the risk of recurrent injury and the development of chronic ankle instability (CAI) [7–9]. Re-injury rates following an initial ankle sprain are high, with recurrent sprains in athletes being reported at over 80% [7]. Meanwhile, CAI can lead to reduced physical activity due to persistent ankle pain, swelling, crepitus, stiffness, weakness, and instability [10], as well as the development of post-traumatic ankle osteoarthritis [2,11,12].

One of the aims of ankle sprain management is to prevent re-injury through enhancing the mechanical and functional stability around the ankle. Mechanical ankle instability relates to excessive joint motion [13], while functional ankle instability is associated with the feeling of the ankle “giving way” [14] due to sensorimotor deficits [15]. These types of instability are frequently managed with therapeutic exercise (to increase ankle joint motion, increase the strength, coordination and postural control around the ankle) and sports-specific activities. Recent clinical guidelines, however, suggest that there is only weak evidence to support the effectiveness of these interventions in preventing future ankle sprains, and therefore CAI [2,16].

Clinical guidelines demonstrate that many forms of treatment exist for ankle sprains, and these vary in their effectiveness [2,16]. However, some contemporary interventions which are used clinically, such as Kinesiotape (KT), are absent from recently published clinical guidelines. KT is an umbrella term used to describe the growing number of elastic adhesive tape varieties used in the prevention and management of sports and musculoskeletal injuries [17]. While several brands of KT exist, their proposed effects are similar, with only subtle variations in their physical properties. Clinically, KT is used as an alternative to the more established taping and bracing techniques, for the prophylaxis and treatment of ankle sprains [18]. While traditional taping techniques have used rigid tape to enhance stability, KT offers an elastic alternative that may be better tolerated and cost effective. According to the manufacturers, KT is latex free, water resistant and can remain in situ for up to 5 days [19]. KT may also assist in ankle sprain management by reducing pain, altering muscle function, improving circulation, enhancing proprioception, and repositioning subluxed joints [17].

At present there is conflicting evidence regarding the effectiveness of KT in the prevention and management of sports injuries and musculoskeletal injuries, as evidenced by five recently published systematic reviews on this area [20–24]. Four systematic reviews reported that KT had little clinical significance or effect on ankle movement and various measures of strength (e.g. isometric, isokinetic, muscle activity), in the long term, compared to usual care or sham tape [20,22–24]. However, in one systematic review, it was found that KT produced an immediate reduction in pain [20], whereas another review reported small improvements in movement and muscle activity [21]. Likely proprioceptive benefits have also been reported [21]. Conflicting findings from these systematic reviews may be explained by (a) insufficient volume of evidence; (b) lack of high quality evidence; and (c) heterogeneity of participants, interventions and outcome measures, since these systematic reviews included evidence from participants with a range of musculoskeletal disorders, spanning the upper and lower limbs, and the spine, as well as neurological and lymphatic conditions. None of these systematic reviews contained evidence on the effect of KT for lateral ankle sprains.

Until recently, the body of evidence evaluating the efficacy of KT in relation to a single injury or musculoskeletal condition has been small. This has precluded a targeted systematic review of the effects of KT at a particular joint, such as the ankle, or a specific diagnosis, such as a lateral ankle sprain. However, a growing body of literature on the use of KT at the ankle over the past five years makes it timely to address some of the limitations of previous systematic reviews. This systematic review, therefore, aimed to identify, evaluate and synthesise evidence relating to the effect of KT applied to the lateral aspect of the ankle, on disability outcomes relevant to lateral ankle sprains.

## Materials and Methods

This systematic review has been written to the Preferred Reporting Items for Systematic Reviews and Meta-Analyses (PRISMA) Statement [25].

### Search Strategy

A comprehensive and systematic electronic database search was performed on April 12, 2014. Seven electronic databases (Cochrane Library, Cumulative Index to Nursing and Allied Health Literature (CINAHL), MEDLINE, Physiotherapy Evidence Database (PEDro), Scopus, SPORTDiscus and Web of Science) were searched for relevant studies with no limits on date or language imposed. Key terms that were inputted into the title and abstract, or keyword fields of databases included “kinesio tap*” and “ankle” (Table 1). Preliminary searching revealed that expansion of search terms to include “kinesio tap*” or “kinesiotap*” or “kinesio-tap*” or “kinaesthetic tap*” or “k tap*” or “kt” or “elastic tap*” and “ankle” did not identify additional, relevant studies. Similarly, no additional primary studies were identified when “ankle” was used as a Medical Subject Heading (MeSH) as well as a keyword. Secondary searching of reference lists of included studies and all systematic reviews identified on KT was also performed to identify additional, relevant studies.

**Table 1:** Search strategy.

Databases	Date range	Key words	Fields
Cochrane Library	1999–2014	(Kinesio tap*) AND (ankle)	Title, abstract, keywords
Cumulative Index to Nursing and Allied Health Literature (CINAHL)	1937–2014	(kinesio tap*) AND (ankle m.p, OR ankle)	Title, abstract, subject headings
MEDLINE	1949–2014	(kinesio tap*) AND (ankle m.p, OR ankle)	Title, abstract, subject headings
Physiotherapy Evidence Database (PEDro)	1929–2014	(Kinesio tap*) AND (ankle)	Abstract and title
Scopus	1966–2014	(Kinesio tap*) AND (ankle)	Article title, abstract, keywords
SPORTDiscus	1900–2014	(Kinesio tap*) AND (ankle)	
Web of Science	1999–2014	(Kinesio tap*) AND (ankle)	Topic

### Study Selection

One researcher (B.W) was involved in the selection of primary studies through review of the title and abstract, and if required, the full text version of the papers. To be eligible for inclusion, studies needed to:

1. Be published in full so that the methodological quality of the study could be assessed. Abstracts of poster presentations and conference presentations were considered insufficient due to their short word limit, which can result in the omission of key information [26].
2. Use a quantitative methodology, with level of evidence classified as II-IV on the National Health and Medical Research Council (NMHRC) Hierarchy of Evidence for intervention studies [27]. This strategy increased the volume of evidence, in the absence of a body of evidence using a randomised controlled trial (RCT) methodology [28,29]. This is particularly relevant in reviews of emerging interventions [30], such as KT. Single case studies were excluded due to their low level of evidence [31].
3. Evaluate the effectiveness of KT applied at the ankle. KT needed to be applied in a manner that was consistent with or inclusive of the technique described by Dr Kenzo Kase for the management of lateral ligament ankle sprains [17]. It was not deemed relevant to exclude studies based on the brand of KT used.
4. Either (a) compare KT to other taping conditions, including rigid tape, placebo tape or no tape or, (b) use a repeated measures design to determine the effect of KT over time.
5. Include participants with a diagnosis of ankle instability, or healthy participants. Studies examining participants with other musculoskeletal, lymphatic or neurological conditions were excluded, as these conditions may impact on the effect of KT.
6. Assess outcomes in terms of disability. Disability was defined as an impairment, activity limitation or participation restriction as per the International Classification of Functioning, Disability and Health (ICF) model [32]. This approach was based on that used by Bialocerkowski et al. (2009) in their recent systematic review [33].

### Quality Assessment

Two researchers (B.W and A.B) independently assessed the quality of the included studies by determining their level of evidence and evaluating their methodological rigour. Any disagreements were discussed until consensus was achieved.

#### Levels of Evidence

The NHMRC’s Hierarchy of Evidence [27] was used to categorise the level of evidence of studies (Table 2). This indicated the potential level of bias present in studies due to their methodological design [31].

**Table 2:** National Health and Medical Research Council Hierarchy of Evidence [27].

Level	Definition
I	A systematic review of level II studies.
II	A randomised controlled trial.
III-1	A pseudorandomised controlled trial (alternate allocation or some other method).
III-2	A comparative study with concurrent controls (non-randomised experimental trial, cohort study, case-control study or interrupted time series with a control group).
III-3	A comparative study without concurrent controls (historical control study, two or more single arm study or interrupted time series without a parallel control group).
IV	Case series with either post-test or pre-test/post-test outcomes.

#### Methodological Rigour

The methodological rigour of studies was assessed to provide an indication of the degree of bias in results [34]. The McMaster Critical Appraisal Tool for Quantitative Studies [35] was used as it is applicable to all types of quantitative study designs [36]. A numerical score of quality was given for each of the 16 items where, if the criterion for an item was met, a score of “one” was given. Alternatively, a score of “zero” was given when the criterion was not fulfilled or only partially fulfilled. Item scores were then summated to provide a score from a maximum of 16, with 16 indicating excellent methodological rigour [37]. Total scores were then divided into five arbitrary categories to reflect the overall methodological rigour of the study: poor (≤8), fair (9–10), good (11–12), very good (13–14) and excellent (15–16) [31]. In addition, five criteria from the PEDro scale (items 3,4,7–9) [38] were used to further explore potential sources of bias in studies which utilised a RCT design.

### Data Extraction and Syntheses

Descriptive statistics were used to summarise the number of included studies, their level of evidence, and methodological rigour. The level of agreement between reviewers when scoring methodological rigour was analysed through a percentage of agreement and calculation of a Kappa statistic and 95% confidence interval (95%CI). Two researchers (B.W, A.B) then independently extracted information from studies. This data were compared, and disagreements resolved by discussion. Extracted data included participant characteristics, intervention parameters, comparison interventions (as applicable), outcome measures, and findings in relation to disability at different time frames.

The effect of KT was expressed as between group mean differences (and 95% confidence intervals) for controlled studies and within group mean differences (and 95% confidence intervals) for case series articles. If findings were not reported in primary studies in this manner, the Physiotherapy Evidence Database confidence interval calculator was utilised to translate findings into this format [39], where adequate data were provided. Post treatment means and standard deviations were used to calculate outcomes for controlled studies, and pre and post treatment means and standard deviations were used to calculate outcomes for case series articles. Findings were considered statistically significant if the 95% confidence interval did not cross “zero” [40].

Findings were also categorised and grouped according to the ICF component of disability [32], and timing of reassessment. For example, outcomes focused on impairments were grouped together, versus those focusing on activity limitations and participation restrictions. An “immediate” effect was defined as the outcome measured directly after the application of KT, and a “short term” effect as an outcome measured up to 24 hours after KT application. A “medium term” effect was considered to be an outcome measured between 24 hours and 1 week, and a “long term” outcome measured more than 1 week [34,41].

The clinical heterogeneity of the primary studies was ascertained by comparing participants, interventions, comparison interventions, outcome measures, and time points of evaluation. It was anticipated that differences would exist which would preclude a meta-analysis, especially given inclusion of variable methodological designs and participants [42]. Included study findings, therefore, were synthesised narratively and interpreted in terms of their methodological rigour.

## Results

### Search Results

**Fig 1:**
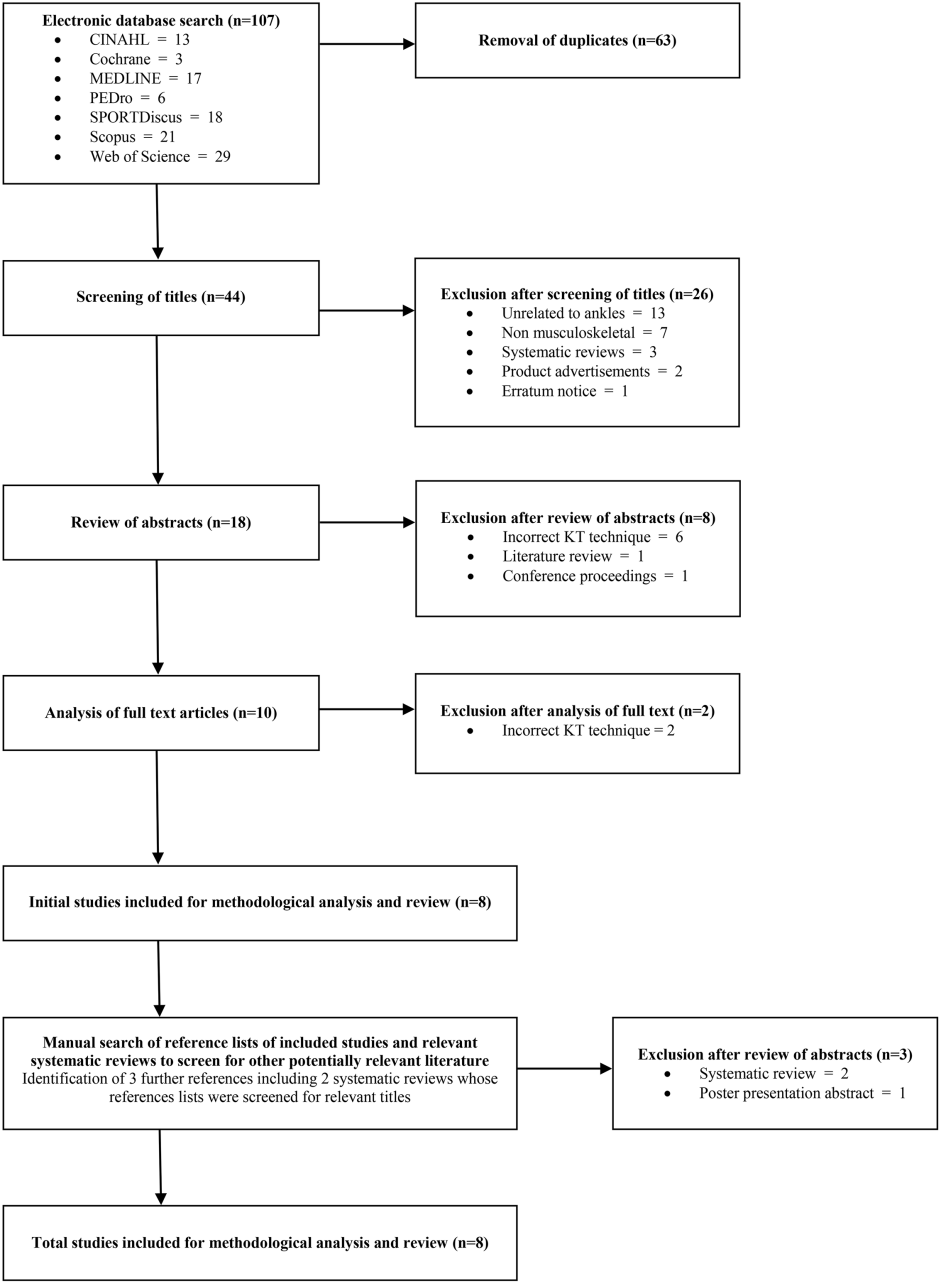
Results of the literature search.

A total of 107 “hits” were gained from the database searching and a further three articles were identified by secondary searching. The majority of articles that were excluded were due to being a duplicate (n = 63) and not related to the ankle (n = 13). Other reasons for exclusion included not being level II-IV evidence (n = 9), including participants with non-musculoskeletal disorders (n = 7), not using the technique described by Kase et al. (2003) [17] (n = 8) and being published in an abstract form (n = 2). A total of eight studies were included in this review, of which seven were published in the last five years [43–50] (Fig 1).

### Quality Assessment

#### Levels of Evidence

The majority of included studies were RCTs (level II evidence) [43, 45–47], whereas the remaining studies used a pseudo-RCT [44], case control [48], cohort [49] (level III evidence) and case series [50] (level IV evidence) methodologies.

#### Methodological Rigour

There was 91% agreement (K = 0.772 (95% CI: 0.650–0.893)) between the reviewers on the scores gained from the Critical Review Form Quantitative Studies. This represents substantial agreement [51]. Consensus was gained on the 12 disagreements. Methodological rigour scores ranged from 7 to 13 from a maximum of 16. Most studies (n = 7) were rated as either good [44,47–50] or very good quality [43,46] (Table 3).

**Table 3:** Methodological rigour of the included studies using the McMaster Critical Review Form for Quantitative Studies [35,36] and the PEDro Scale [38].

	Criterion—Critical Review Form	Bicici et al, 2012 [43]	Briem et al, 2011 [44]	Fayson et al, 2013 [50]	Halseth et al, 2014 [45]	Nakajima et al, 2013 [46]	Semple et al, 2012 [47]	Shields et al, 2013 [49]	Simon et al, 2014 [48]	Total
1	Purpose clearly stated	1	1	1	0	1	1	1	0	6
2	Literature review relevant	1	1	1	1	1	1	1	1	8
3	Study design appropriate to study aims	1	1	1	1	1	1	1	0	7
4	No biases present	0	0	0	0	0	0	0	0	0
5	Sample described in detail	1	1	1	0	1	1	1	1	7
6	Sample size justified	0	0	0	0	0	0	0	0	0
7	Informed consent gained	1	1	1	0	1	1	1	1	7
8	Validity of outcome measures used	0	0	0	0	0	0	0	0	0
9	Reliability of outcome measures used	1	0	0	0	1	1	0	1	4
10	Intervention described in detail	1	1	1	1	1	1	1	1	8
11	Statistical reporting of results	1	1	1	1	1	1	1	1	8
12	Appropriate statistical analysis	1	1	1	1	1	1	1	1	8
13	Clinical importance reported	1	1	1	1	1	1	1	1	8
14	Appropriate conclusions	1	1	1	1	1	1	1	1	8
15	Clinical implications reported	1	1	1	0	1	1	1	1	7
16	Study limitations acknowledged	1	1	1	0	1	0	1	1	6
	**Total**	**13**	**12**	**12**	**7**	**13**	**12**	**12**	**11**	
	**Descriptor [31]**	Very Good	Good	Good	Poor	Very Good	Good	Good	Good	
	**Criterion—PEDro scale**									
3	Allocation was concealed	na			x	na	na			
4	Groups were similar at baseline	✓			✓	✓	X			
7	Blinding of assessors	x			X	x	x			
8	Obtained at least 85% of measures from at least one key outcome	✓			✓	✓	✓			
9	Intention to treat analysis performed	X			X	✓	X			

1 = criteria fulfilled completely, 0 = criteria not fulfilled completely

Quality category: poor (≤8), fair (9–10), good (11–12), very good (13–14), and excellent (15–16) [31],

✓ = criterion fulfilled, x = criterion not fulfilled, na not appropriate

The strengths of the included studies, based on the assessment of methodological rigour, included a clear justification of the need for the study (item 1), provided a detailed description of the intervention used (item 10), conducted appropriate statistical analyses to address the study aim (item 12), discussed the clinical importance of their results (item 13) and derived appropriate conclusions from the data (item 14) (Table 3). The majority of studies also contained an aim which was clearly written (item 1), used an appropriate method to address the study aim (item 3), described their sample in detail (item 5), gained informed consent from their participants (item 7), discussed the clinical implications of their results (item 15), and acknowledged the major limitations of their study (item 16). A paucity of evidence was found on the validity (item 8) and reliability (item 9) of outcome measures used to measure the effect of KT. None of the studies provided a justification of their sample size (item 6) or controlled for major biases (item 4) (Table 3).

There was also excellent agreement between reviewers on the scores gained from the five PEDro criteria (95% agreement, k = 0.89 (95%CI: 0.69–1.0)) across the four RCTs. The one disagreement was resolved by discussion between researchers. As shown in Table 3, the main sources of identified bias were due to the lack of blinding of assessors (criterion 7) and lack of confidence that all participants received the interventions as allocated (intention to treat) (criterion 9).

### Description of Included Studies

Table 4 provides a summary of the population, intervention, comparison, and outcome measures for the included studies. A total of 276 participants were included in the eight studies. Four out of the eight studies included participants with ankle instability (n = 84) [43,44,48,49]. Data on the effect of KT on healthy ankles was gained in seven studies, from 192 participants [44,45–50]. The average sample size was 34 (range = 15–60) [42,48]. There were no reported instances of participant attrition.

**Table 4:** Study characteristics.

Study	Design	Level of Evidence^a^	Objective(s)	Participant Characteristics	KT and Comparison	Outcome Measures [Timing]
Studies including participants with unstable ankles						
Bicici et al, 2012 [43]	RCT (Cross over)	II	Effect of KT, rigid tape, and placebo tape compared to no tape on postural control, muscle endurance and functional task performance in athletes with unstable ankles	15 basketball players with chronic inversion ankle sprains (>3) and a diagnosis of functional ankle instability as determined by the CAIT **Gender:** male (n = 15) **Age** (years)*: 20.33 (1.4)	**KT:** KT of the tibiofibular ligament and peroneus longus and brevis muscles **Comparison:** rigid tape for lateral ligament ankle sprain, placebo tape via ‘I’ shaped rigid tape strips applied with no tension, and no tape	**Body Function / Structure:** postural control, muscle endurance [immediately after KT application] **Activity and Participation:** functional task performance [immediately after KT application]
Briem et al, 2011 [44]	Pseudo-RCT	III-1	Effect of KT and rigid tape compared to no tape on peroneus longus muscle activity and perceived stability during an inversion perturbation in athletes with stable and unstable ankles	30 premier league athletes (soccer, handball, basketball); 15 with ankle instability, 15 with no ankle instability **Gender:** male (n = 30), **Age** (years)*: 24.5 (5.0)	**KT:** KT of peroneus longus **Comparison:** rigid tape for lateral ligament ankle sprain, and no tape	**Body Function / Structure:** muscle activity, perceived stability [immediately after KT application]
Simon et al, 2014 [48]	Case-control	III-2	Effect of KT on proprioception in participants with unstable ankles compared to healthy controls	28 participants **KT group:** unstable ankles (n = 14) **Gender:** male (n = 9), female (n = 6) **Age** (years)*: 20.8 (1.4) **Control group:** healthy participants) (n = 14) **Gender:** male (n = 2), female (n = 12) **Age** (years)*: 21.2 (2.6)	**KT:** KT for lateral ligament ankle sprain**Comparison:** no tape	**Body Function / Structure:** proprioception [immediate, 72 hours after KT application]
Shields et al, 2013 [49]	Cohort	III-2	Immediate and lasting effects of KT on postural control in healthy, coper and unstable ankles	60 participants stratified into equal groups (n = 20) of healthy, coper or unstable ankles by history of ankle injury and CAIT scores **Gender:** male (n = 25), female (n = 35) **Age** (years)*: 21.5 (2.6)	**KT:** KT for lateral ligament ankle sprain Comparison: no tape	**Body Function / Structure:** postural control [immediate, 24 hours after KT application]
Studies including participants with stable ankles						
Fayson et al, 2013 [50]	Case series	IV	Effect of KT on ankle joint stiffness and functional task performance in healthy participants	30 healthy participants **Gender:** female (n = 30) **Age** (years)*: 20.4 (1.0)	**KT:** KT for lateral ligament ankle sprain Comparison: no tape	**Body Function / Structure:** ankle joint stiffness [immediate, 24 hours after KT application] **Activity and Participation:** functional task performance [immediate, 24 hours after KT application]
Halseth et al, 2004 [45]	RCT(Cross over)	II	Effect of KT compared to no tape on proprioception in healthy participants	30 healthy participants **Gender:** male (n = 15), female (n = 15) **Age** (years)**: 18–30	**KT:** KT for lateral ligament ankle sprain **Comparison:** no tape	**Body Function / Structure:** proprioception [immediately after KT application]
Nakajima et al, 2013 [46]	RCT (Parallel)	II	Effect of KT compared to sham KT on dynamic postural control and functional task performance in healthy participants	52 healthy participants **Gender:** male (n = 28), female (n = 24) **Age** (years)*: 22.12 (2.08)	**KT:** KT for lateral ligament ankle sprain with tension **Comparison:** KT for lateral ligament ankle sprain without tension (sham)	**Body Function / Structure:** postural control [immediate, 24 hours after KT application] **Activity and Participation:** functional task performance [immediate, 24 hours after KT application]
Semple et al, 2012 [47]	RCT (Cross over)	II	Effect of KT compared to no tape on postural control in healthy athletes	31 healthy, semi-professional rugby union players **Gender:** male (n = 31) **Age** (years)*: 19.57 (0.76)	**KT:** Pre-cut KT for lateral ligament ankle sprain **Comparison:** no tape	**Body Function / Structure:** postural control [immediately after KT application]


^a^ Level of Evidence as per the Hierarchy of Evidence [27]

KT kinesiotape

CAIT Cumberland Ankle Instability Tool

*values represent Mean (standard deviation)

** values represent range

The participants in all studies were young (<31 years of age). There were more male (n = 155) than female participants (n = 121). Athletic participants (basketball [43,44], handball [44], rugby union [47], and soccer [44]) were used in three studies.

While all studies investigated the effects of KT compared to no tape, one study compared KT to a sham KT condition [46] where no tension was applied to the tape during its application. Furthermore, KT was compared to rigid tape in two studies [43,44] with one of these studies also including a placebo rigid taping condition for comparison [43] (Table 4).

All studies investigated the effect of KT on impairment variables [43–50]. Ten different outcome measures were used to evaluate six impairment variables (postural control, proprioception, ankle joint stiffness, muscle activity of fibularis longus, isotonic muscle endurance of the plantarflexors, and perceived stability). The Star Excursion Balance Test (SEBT) was the only outcome measure used in more than one study to evaluate impairments [43,46]. In addition to assessing impairments, three studies also evaluated effect of KT on activity limitations and participation restrictions [43,46,50], by using measures of functional task performance. Four different outcome measures were used across these studies. Vertical jump height was the only outcome measure that was used in more than one study, however the methodology employed was not homogenous [43,46]. In every study, outcomes were evaluated immediately following application of KT [43–50]. In addition, outcomes were evaluated at 24 hours after KT application in three studies [46,49,50]. Simon et al (2014) [48] also assessed outcomes at 72 hours after KT application (Table 4).

These results demonstrate that there was clinical heterogeneity between the studies in their samples (unstable ankles and healthy ankles, athletes and non-athletes), comparison interventions (rigid tape, placebo/sham tape, no tape,), outcome measures (various measures of impairment, activity limitation/participation restriction), and time points of evaluation (immediate, 24 and 72 hours post KT application). While this heterogeneity precluded a meta-analysis, a narrative synthesis of the findings from the studies was possible.

### Syntheses of Findings

A beneficial effect was demonstrated in five of the eight studies [43,46–49]. No study reported adverse effects associated with the use of KT.

### Measures of Impairment of Body Structure or Body Function.

#### Postural Control

Four studies examined the effect of KT on postural control [43,46,47,49] which was evaluated using various outcome measures [eg. SEBT, Biodex Balance System (BBS), Kinesthetic Ability Trainer Test (KATT), and Time to Boundary (TTB) and Centre of Pressure (COP) during single leg stance]. One very good quality, level II study examined the effect of KT on unstable ankles and found and increase in static balance in the KT group compared to no tape [43]. Shields et al. (2013) [49] (a good quality, level III-2 study) found increased postural control (in the medial/lateral plane during the time to boundary test) after KT application in participants with ankle sprains [49]. These small changes were not present in participants without a history of ankle sprain or those who had a history of ankle sprain but report no further dysfunction [49]. In two studies conducted solely on healthy participants, KT was found to immediately increase postural control in females (increased SEBT scores for medial and posteromedial directions) [46] and rugby players, particularly those who played in the forwards [47] (Table 5). Both of these studies were of good [47] to very good quality [46], level II evidence (Table 3).

**Table 5:** Effectiveness of KT compared to standard tape, placebo/sham tape and no tape in people with stable and unstable ankles.

**ICF Level**	**Variable**	**Study**	**Population [Sample Size]**	**Intervention and Comparison**	**Outcome measure [units]**	**Additional outcome measure information**	**Timing**	**Mean difference [95% confidence interval]**
Body function	Postural control	Bicici et al, 2012 [43]	Basketballers with chronic ankle inversion sprains [n = 15]	KT versus standard tape	Kinesthetic Ability Training Test—static balance test score		Immediate	-78.6 [-234.5 to 77.3]
				KT versus placebo tape	Kinesthetic Ability Training Test—static balance test score		Immediate	-127.13 [-275.67 to 21.41]
				KT versus no tape	Kinesthetic Ability Training Test—static balance test score		Immediate	-151.2 [-301.16 to -1.24]*
				KT versus standard tape	Kinesthetic Ability Training Test—dynamic balance test score		Immediate	18.0 [-426.12 to 462.12]
				KT versus placebo tape	Kinesthetic Ability Training Test—dynamic balance test score		Immediate	-153.0 [-549.69 to 243.69]
				KT versus no tape	Kinesthetic Ability Training Test—dynamic balance test score		Immediate	-172.0 [-559.86 to 215.86]
				KT versus standard tape	SEBT (cm)	Anterior	Immediate	-0.09 [-3.27 to 3.11]
				KT versus standard tape	SEBT (cm)	Anteromedial	Immediate	-0.08 [-2.62 to 2.46]
				KT versus standard tape	SEBT (cm)	Medial	Immediate	0.53 [-5.99 to 7.05]
				KT versus standard tape	SEBT (cm)	Posteriomedial	Immediate	0.40 [-5.25 to 6.05]
				KT versus standard tape	SEBT (cm)	Posterior	Immediate	0.39 [-4.24 to 5.02]
				KT versus standard tape	SEBT (cm)	Posterolateral	Immediate	0.07 [-5.00 to 5.14]
				KT versus standard tape	SEBT (cm)	Lateral	Immediate	-0.73 [-5.31 to 3.85]
				KT versus standard tape	SEBT (cm)	Anterolateral	Immediate	0.10 [-4.81 to 4.38]
				KT versus placebo tape	SEBT (cm)	Anterior	Immediate	0.32 [-2.94 to 3.58]
				KT versus placebo tape	SEBT (cm)	Anteromedial	Immediate	1.59 [-0.88 to 4.06]
				KT versus placebo tape	SEBT (cm)	Medial	Immediate	0.03 [-6.37 to 6.43]
				KT versus placebo tape	SEBT (cm)	Posteriomedial	Immediate	-0.05 [-5.54 to 5.44]
				KT versus placebo tape	SEBT (cm)	Posterior	Immediate	0.28 [-4.21 to 4.77]
				KT versus placebo tape	SEBT (cm)	Posterolateral	Immediate	-0.20 [-5.36 to 4.96]
				KT versus placebo tape	SEBT (cm)	Lateral	Immediate	0.27 [-4.15 to 4.69]
				KT versus placebo tape	SEBT (cm)	Anterolateral	Immediate	0.19 [-4.03 to 4.41]
				KT versus no tape	SEBT (cm)	Anterior	Immediate	0.42 [-2.84 to 3.68]
				KT versus no tape	SEBT (cm)	Anteromedial	Immediate	0.88 [-2.17 to 2.33]
				KT versus no tape	SEBT (cm)	Medial	Immediate	-0.05 [-6.93 to 6.83]
				KT versus no tape	SEBT (cm)	Posteriomedial	Immediate	0.48 [-5.38 to 6.34]
				KT versus no tape	SEBT (cm)	Posterior	Immediate	0.07 [-4.86 to 5.00]
				KT versus no tape	SEBT (cm)	Posterolateral	Immediate	0.07 [-5.06 to 5.20]
				KT versus no tape	SEBT (cm)	Lateral	Immediate	0.34 [-4.17 to 4.85]
				KT versus no tape	SEBT (cm)	Anterolateral	Immediate	0.06 [-4.35 to 4.47]
		Nakajima et al, 2013 [46]	Healthy participants [n = 52]	KT versus sham tape	SEBT (cm)		Immediate	^a^
				KT versus sham tape	SEBT (cm)		24 hours	^a^
		Semple et al 2012 [47]	Healthy rugby players [n = 31]	KT versus no tape	Posture stability using BBS (index)	Overall index	Immediate	-0.07 [-1.44 to 0.04]
				KT versus no tape	Posture stability using BBS (index)	Anterioposterior	Immediate	-0.40 [-0.98 to 0.18]
				KT versus no tape	Posture stability using BBS (index)	Medial/lateral	Immediate	-0.40 [-0.73 to -0.07]*
		Shields et al, 2013 [49]	Healthy [n=20], coper [n = 20], unstable ankles [n = 20]	KT versus no tape	SLB using force plate	TTB (s)	Immediate	^b^
				KT versus no tape	SLB using force plate	TTB (s)	24 hours	^c^
				KT versus no tape	SLB using force plate	CoP (m)	Immediate	^b^
				KT versus no tape	SLB using force plate	CoP (m)	24 hours	^c^
	Proprioception	Halseth et al, 2004 [45]	Healthy participants [n = 30]	KT versus no tape	RJJS (°)	Absolute error (PF)	Immediate	-0.12 [-0.69 to 0.45]
				KT versus no tape	RJJS (°)	Absolute error (PF/INV)	Immediate	0.08 [-0.38 to 0.54]
				KT versus no tape	RJJS (°)	Constant error (PF)	Immediate	0.36 [-0.62 to 1.34]
				KT versus no tape	RJJS (°)	Constant error (PF/INV)	Immediate	-0.26 [-1.11 to 0.59
		Simon et al, 2014 [48]	Unstable ankles [n = 15], unstable ankles [n = 15]	KT versus no tape	Everson force sense (N)		Immediate	1.2 [0.20 to 2.20]*
				KT versus no tape	Everson force sense (N)		72 hours	0.7 [-0.07 to 1.49]
	Ankle joint stiffness	Fayson et al, 2013 [48]	Healthy participants [n = 30]	KT versus no tape	TCJ anterior translation (mm)		Immediate	0.98 [-0.47 to 2.43] (MAX))
				KT versus no tape	TCJ anterior translation (mm)		Immediate	-2.95 [-8.10 to 2.20] (25Nm)**
				KT versus no tape	TCJ anterior translation (mm)		Immediate	-5.89 [-12.69 to 0.91] (50Nm)**
				KT versus no tape	TCJ anterior translation (mm)		Immediate	-2.85 [-6.45 to 0.75] (75 Nm)
				KT versus no tape	TCJ anterior translation (mm)		Immediate	-1.79 [-5.52 to 1.96] (100 Nm)
				KT versus no tape	TCJ anterior translation (mm)		Immediate	1.22 [-3.50 to 5.94] (125 Nm)
				KT versus no tape	TCJ anterior translation (mm)		24 hours	0.24 [-1.28 to 1.76] (MAX)
				KT versus no tape	TCJ anterior translation (mm)		24 hours	-3.57 [-11.39 to 4.25] (25 Nm)**
				KT versus no tape	TCJ anterior translation (mm)		24 hours	-1.93 [-5.98 to 2.12] (50Nm)
				KT versus no tape	TCJ anterior translation (mm)		24 hours	-3.02 [-7.71 to 1.67] (75Nm)
				KT versus no tape	TCJ anterior translation (mm)		24 hours	-0.64 [-4.33 to 3.05] (100Nm)
				KT versus no tape	TCJ anterior translation (mm)		24 hours	2.28 [-8.09 to 3.53] (125Nm)
	Muscle activity	Briem et al, 2011 [44]	Athletes: Unstable ankles [n = 15], stable ankles [n = 15]	KT versus standard tape	Fibularis longus mean activation (%MVC)		Immediate	^d^
	Muscle endurance	Bicici et al, [43]	Basketballers with chronic ankle instability [n = 15]	KT versus standard tape	Standing heal test (n)		Immediate	4.87 [0.92 to 8.82]*
				KT versus placebo tape	Standing heal test (n)		Immediate	1.8 [-2.49 to 6.09]
				KT versus no tape	Standing heal test (n)		Immediate	1.8 [-2.72 to 6.32]
	Perceived stability	Briem et al [44]	Athletes: Unstable ankles [n = 15], stable ankles [n = 15]	KT versus standard tape	Participants questioned on the most stable and least stable position		Immediate	^e^
Activity / participation	Functional task performance	Bicici et al 2012 [43]	Basketballers with chronic ankle inversion sprains [n = 15]	KT versus standard tape	Hopping test (s)		Immediate	0.06 [-0.41 to 0.53]
				KT versus placebo tape	Hopping test (s)		Immediate	-0.39 [-0.801 to 0.02]
				KT versus no tape	Hopping test (s)		Immediate	-0.59 [-0.94 to -0.24]*
				KT versus standard tape	Hurdle test (s)		Immediate	-0.09 [-0.41 to 0.25]
				KT versus placebo tape	Hurdle test (s)		Immediate	-0.24 [-0.56 to 0.08]
				KT versus no tape	Hurdle test (s)		Immediate	-0.33 [-0.64 to -0.27]*
				KT versus standard tape	Vertical jump height (cm)		Immediate	3.46 [1.74 to 5.18]*
				KT versus placebo tape	Vertical jump height (cm)		Immediate	0.71 [-0.96 to 2.38]
				KT versus no tape	Vertical jump height (cm)		Immediate	0.54 [-1.11 to 2.19]
		Fayson et al, 2013 [50]	Healthy women [n = 30]	KT versus no tape	Time to stabilise during hopping tasks (sec)	Forward Fx	Immediate	0.00 [-0.55 to 0.55]
				KT versus no tape	Time to stabilise during hopping tasks (sec)	Forward Fy	Immediate	0.00 [-0.91 to 0.91]
				KT versus no tape	Time to stabilise during hopping tasks (sec)	Forward Fz	Immediate	0.30 [-0.79 to 1.39]
				KT versus no tape	Time to stabilise during hopping tasks (sec)	Backward Fx	Immediate	-0.10 [-0.55 to 0.35]
				KT versus no tape	Time to stabilise during hopping tasks (sec)	Backward Fy	Immediate	-0.30 [-1.06 to 0.46]
				KT versus no tape	Time to stabilise during hopping tasks (sec)	Backward Fz	Immediate	-0.81 [-1.83 to 0.23]
				KT versus no tape	Time to stabilise during hopping tasks (sec)	Medial Fx	Immediate	0.00 [-0.79 to 0.79]
				KT versus no tape	Time to stabilise during hopping tasks (sec)	Medial Fy	Immediate	-0.4 [-0.94 to 0.14]
				KT versus no tape	Time to stabilise during hopping tasks (sec)	Medial Fz	Immediate	-0.3 [-1.32 to 0.72]
				KT versus no tape	Time to stabilise during hopping tasks (sec)	Lateral Fx	Immediate	0.10 [-0.73 to 0.93]
				KT versus no tape	Time to stabilise during hopping tasks (sec)	Lateral Fy	Immediate	0.10 [-0.26 to 0.46]
				KT versus no tape	Time to stabilise during hopping tasks (sec)	Lateral Fz	Immediate	0.50 [-0.75 to 1.75]
				KT versus no tape	Time to stabilise during hopping tasks (sec)	Forward Fx	24 hours	0.00 [-0.57 to 0.57]
				KT versus no tape	Time to stabilise during hopping tasks (sec)	Forward Fy	24 hours	0.20 [-0.71 to 1.11]
				KT versus no tape	Time to stabilise during hopping tasks (sec)	Forward Fz	24 hours	0.20 [-0.93 to 1.33]
				KT versus no tape	Time to stabilise during hopping tasks (sec)	Backward Fy	24 hours	0.00 [-0.43 to 0.43]
				KT versus no tape	Time to stabilise during hopping tasks (sec)	Backward Fx	24 hours	0.20 [-1.03 to 1.43]
				KT versus no tape	Time to stabilise during hopping tasks (sec)	Backward Fz	24 hours	0.30 [-1.33 to 0.73]
				KT versus no tape	Time to stabilise during hopping tasks (sec)	Medial Fx	24 hours	0.40 [-0.42 to 1.22]
				KT versus no tape	Time to stabilise during hopping tasks (sec)	Medial Fy	24 hours	-0.60 [-1.17 to -0.03]*
				KT versus no tape	Time to stabilise during hopping tasks (sec)	Medial Fz	24 hours	-0.20 [-1.26 to 0.86]
				KT versus no tape	Time to stabilise during hopping tasks (sec)	Lateral Fx	24 hours	0.20 [-0.61 to 1.01]
				KT versus no tape	Time to stabilise during hopping tasks (sec)	Lateral Fy	24 hours	0.20 [-0.15 to 0.55]
				KT versus no tape	Time to stabilise during hopping tasks (sec)	Lateral Fz	24 hours	0.2 [-1.20 to 1.60]
		Nakajima et al, 2013 [46]	Healthy participants [n = 52]	KT versus sham tape	Vertical jump height (cm)		Immediate	^f^
				KT versus sham tape	Vertical jump height (cm)		24 hours	^f^

* statistically significant between group difference

** Statistically significant result reported by the author

^a^ insufficient information to calculate mean difference and 95% confidence interval. Authors reported a statistically significant increase in postural control in females in the medial and posteromedial directions immediately after KT application, compared to the placebo tape group

^b^ insufficient information to calculate mean difference and 95% confidence interval. No results reported in the study

^c^ insufficient information to calculate mean difference and 95% confidence interval. Authors reported a statistically significant increase in time to boundary in the medial/lateral plane in the KT group compared to copers and healthy participants

^d^ insufficient information available to calculate mean difference and 95% confidence interval. Authors reported no statistically significance difference in EMG fibularis longus activity in the KT group compared with no tape, during an inversion perturbation

^e^ not appropriate to calculate mean difference and 95% confidence intervals. Authors reported that those with stable ankles perceived KT to be the most stable taping condition and rigid tape the least stable taping condition. Those with unstable ankles perceived rigid tape to be the most stable taping condition and no tape as the least stable taping condition followed by KT

^f^ Insufficient information available to calculate mean different and 95% confidence interval. Authors reported no statistically significant different in vertical jump height between the taping groups

SEBT Star Excursion Balance Test

BBS Biodex Balance System

CoP Centre of Pressure

TTB Time to Boundary

SLB Single Leg Balance

RJJS Reproduction of Joint Position Sense

MVC maximum voluntary contraction

PF Plantarflexion

PF/INV Plantarflexion and 20 degrees of inversion

TCJ Talocrural Joint

MAX Maximum Displacement

#### Proprioception

Two studies examined the effect of KT on measures of proprioception [45,48]. In their good quality, level II study, Simon et al. (2013) [48] found that after wearing KT for 72 hours, proprioceptive deficits in those with CAI improved to near that of healthy adults. In contrast, Halseth et al. (2004) [45] concluded that KT does not enhance proprioception in healthy adults (Table 5). Despite this study being graded as level II, its methodological quality was rated as poor (Table 3).

#### Ankle Joint Stiffness

One good quality level IV study [48] evaluated the effect of KT on ankle joint stiffness based on force data gained from anterior translation of the talocrural joint in healthy participants. KT had no effect on ankle joint stiffness immediately following application or in the short term (24 hours) (Table 5).

#### Muscle Activity of Fibularis Longus

One good quality, level III study [44] evaluated the effect of KT on peak muscle activity and time to peak muscle activity of fibularis longus using surface electromyography readings during a sudden inversion perturbation in male athletes, with and without unstable ankles. No significant benefit was found for KT compared with standard taping, in either group, immediately following tape application (Table 5).

#### Muscle Endurance of the Plantarflexors

One very good quality, level II study [43] evaluated the effect of KT on isotonic endurance of the plantarflexors using a single leg standing heel raise test in male basketball players with CAI. Compared to rigid tape, KT had a statistically significant, immediate, increase on plantarflexor muscle endurance in basketball players with CAI (Table 5).

#### Perceived Stability

One good quality, level III study [44] evaluated the effect of KT on perceived stability using subjective comparison to other taping conditions during a sudden inversion perturbation in a group of healthy athletic male participants, stratified into stable and unstable ankle groups. While KT was perceived to not be as stable as rigid tape by those with unstable ankles, it was reported as the most stable taping condition by those with healthy ankles (Table 5).

#### Measures of Activity Limitation and Participation Restriction

Three studies [43,46,50] evaluated the effects of KT on activity limitations measured by functional task performance during vertical jump tests [43,46] and a variety of hopping tests [42,49]. The very good quality, level II study by Bicici et al. [43], which recruited participants with CAI, found that KT resulted in immediate, improved performance times during a single limb hurdle and a hop test, compared to no tape, following tape application and or at 24 hours. In addition, vertical jump height was significantly greater in the KT group compared to the standard (rigid) tape group (Table 5).

Two studies evaluated the effects of KT on functional task performance in healthy participants [46,50]. Nakajima et al. (2013), in their very good quality, level II study, found that KT had no effect on vertical jump height, compared to placebo tape. Similarly, Fayson et al. (2013) [50], in their good quality, level IV study, found that KT did not have a statistically significant effect (immediately or at 24 hours) on time to stabilisation measures for a multi-directional hopping test, when compared to no tape (Table 5).

## Discussion

This systematic review is the first to identify, evaluate and synthesise evidence relating to the effect of KT on disability outcomes associated with its application to the lateral aspect of the ankle, in healthy participants and participants with ankle instability. The studies included in this systematic review were typically of good to very good quality and ranged from level II to level IV evidence. Narrative synthesis of the available evidence demonstrated that KT may have different effects on participants with unstable ankles compared to those that are healthy (Table 6). This adds to the body of knowledge on KT and extends the work by Williams et al. (2012) [21].

**Table 6:** Summary of the effect of KT on healthy and unstable ankles.

Variable	Healthy	Unstable
**Body Function**		
Postural control	[46]**, [47]*, [49]*	[43]***
Proprioception	NS	[48]***
Ankle joint stiffness	NS	
Muscle activity—fibularis longus	NS	NS
Muscle endurance—calf		[43]***
**Activity / Participation**		
Hopping	NS	[43]***
Hurdles		[43]***
Vertical jump	NS	[43]*

* compared to rigid tape

** compared to sham tape

*** compared to no tape

NS non-significant results

Our results suggest that in participants with unstable ankles, KT is perceived as providing less stability compared to rigid tape [44]. However, when compared with standard tape, it may increase plantarflexor endurance [43] and increase vertical jump height [43]. Moreover, when compared with no tape, it may decrease proprioceptive deficits [48] and increase the ability to perform activities [43]. This is in contrast to the trends found in healthy participants. Healthy participants reported that KT is the most stable form of taping [44]. Improvements in postural control were found when compared to no tape [47,49] and sham tape [46] (Table 6).

Confidence can be placed in these results due to the rigour of our methods. Seven databases were used to identify primary studies, as well as secondary searching [34]. Although a simplistic search strategy was used, employing a more detailed search did not identify additional studies. Thus, it is unlikely that primary evidence was omitted from this systematic review. All types of quantitative studies were included in this review. This may have introduced bias, however, this approach was considered appropriate considering the limited volume of evidence on this topic [28,31]. Methodological rigour of the primary studies was assessed using an established protocol [31,34,35,40,41], and the results of primary studies interpreted based on these findings. This study specifically addressed the limitations of previous systematic reviews on the effectiveness of KT and focused on synthesising evidence from one anatomical area (the ankle). It therefore is not surprising that the results gained are in contrast to previous reviews [20–24].

A number of hypotheses may explain the observed differences of KT on healthy and unstable ankles. In the presence of impaired proprioception following a lateral ankle sprain [15, 52], it is biologically plausible that KT may increase afferent input and hence improve measures of proprioception [53]. As it is less likely that proprioceptive deficits exist in healthy ankles [54], the application of KT in this population may not result in significant proprioceptive improvements, as baseline measures may already be near to optimal. A similar hypothesis could explain the ability of KT to improve activities in those with unstable ankles but not healthy ankles [55]. Moreover, it is plausible that KT could increase the self efficacy of the individual with an unstable ankle, potentially resulting in greater confidence while performing activities. The healthy individual may not lack confidence with these activities. Hence, the possible psychological benefits of KT may be small, insignificant or not present in healthy individuals.

The ability of KT to alter efferent responses in participants, with unstable ankles, however, is more questionable. KT failed to increase fibularis longus muscle activity [44] and was found not to be effective in improving most measures of postural control in participants with unstable ankles [42]. This is in contrast to its observed effects on healthy participants, where it has been shown to consistently increase measures of postural control [46,47,49]. It should be noted however that these effects were small (e.g. 0.15s improvement in the medial-lateral plane for TTB absolute minima from pretest to 24 hours after taping) [49], demonstrated only by specific outcome measures (e.g. medial and posteromedial directions of the SEBT) [46], and isolated to subgroups of healthy participants (e.g. forward rugby players) [47]. KT alone, therefore, may be insufficient to improve postural control in those with a baseline deficit. With increased afferent input, those without an ankle injury may have a greater capacity to improve on unfamiliar tests such as the SEBT, BBS and single leg stance due to a learning effect [56]. Conversely, those with an unstable ankle may not improve due to the potentially limiting impacts related to mechanical and functional instability that may be present following a lateral ligament ankle sprain. KT may simply not provide the injured participant with enough mechanical support to facilitate improved confidence during performance of postural control tasks, such as the SEBT.

The results of this systematic review, however, must be interpreted with consideration of the low volume of primary studies, clinical heterogeneity in variables that were evaluated in the primary studies, and paucity of psychometric information that underpinned the justification of outcome measures. Eight studies met the inclusion criteria and data from 276 participants formed the basis of our results. None of the studies provided a justification of their sample size so it is unknown whether these studies were underpowered [57,58]. Moreover, little homogeneity existed between the studies regarding how the effect of KT was evaluated. The only outcome measures used in more than one study were the SEBT and vertical jump height. While the test procedure for the SEBT was consistent between two studies, the methods used to determine vertical jump height varied. It was more common for researchers to utilise outcome measures evaluating the effects of KT at the impairment level, compared to activity limitation or participation restriction, despite clinical guidelines recommending that a more holistic evaluation of disability should occur [2]. Importantly, there is no linear relationship between impairments and activity limitations [59], and as such, impairments cannot be used to predict activity limitations or participation restrictions. Furthermore, with a paucity of information provided justifying the psychometric properties of the outcome measures used in the primary studies, it is unknown whether the outcome measures behaved as expected. Future research, therefore, should focus on defining a core set of standardised outcome measures with sound psychometric properties for ankle sprains.

The studies included in this systematic review evaluated the effects of KT in the short to medium term (up to 72 hours post application) despite KT being able to remain in situ for up to 5 days [19]. Thus, the long term benefits of KT are not known, particularly in relation to the prevention of primary and recurrent ankle sprains, and thus should be further researched. Moreover, in this study the effect of KT was determined based on between group mean differences (or within group mean differences) and 95% confidence intervals. It was envisaged that the results could be interpreted in terms of clinical significance, if the between group mean difference was greater than the minimal clinically important difference (MCID) reported in the literature. However, there is a paucity of evidence on the MCID for outcome measures used in the primary studies. Therefore, the clinical significance of the findings is not known [40]. Based on our findings, future studies should therefore be adequately powered, and use psychometrically-sound outcome measures to comprehensively evaluate disability over the long term following KT application.

## Conclusions

Based on the syntheses of results from 8 primary studies, which included a total of 276 participants, it is recommended that KT could be used in clinical practice to prevent lateral ankle injuries through its effects on postural control, and manage lateral ankle injuries, due to its positive effects on proprioception, muscle endurance and activity performance. Future research on the effect of KT on the rate of ankle injury is required to strengthen this recommendation. It must be noted that KT may not provide sufficient mechanical support to unstable ankles to facilitate improved confidence during the performance of postural control tasks. Adverse events associated with KT appear unlikely.
